# Volatile Drosophila Cuticular Pheromones Are Affected by Social but Not Sexual Experience

**DOI:** 10.1371/journal.pone.0040396

**Published:** 2012-07-11

**Authors:** Jean-Pierre Farine, Jean-François Ferveur, Claude Everaerts

**Affiliations:** Centre des Sciences du Goût et de l’Alimentation, Unité Mixte de Recherche 2572-Centre National de la Recherche Scientifique, Unité Mixte de Recherche1324-Institut National de la Recherche Agronomique, Université de Bourgogne, Dijon, France; VIB & Katholieke Universiteit Leuven, Belgium

## Abstract

Recognition of conspecifics and mates is based on a variety of sensory cues that are specific to the species, sex and social status of each individual. The courtship and mating activity of *Drosophila melanogaster* flies is thought to depend on the olfactory perception of a male-specific volatile pheromone, *cis*-vaccenyl acetate (*c*VA), and the gustatory perception of cuticular hydrocarbons (CHs), some of which are sexually dimorphic. Using two complementary sampling methods (headspace Solid Phase Micro-Extraction [SPME] and solvent extraction) coupled with GC-MS analysis, we measured the dispersion of pheromonal CHs in the air and on the substrate around the fly. We also followed the variations in CHs that were induced by social and sexual interactions. We found that all CHs present on the fly body were deposited as a thin layer on the substrate, whereas only a few of these molecules were also detected in the air. Moreover, social experience during early adult development and in mature flies strongly affected male volatile CHs but not *c*VA, whereas sexual interaction only had a moderate influence on dispersed CHs. Our study suggests that, in addition to their role as contact cues, CHs can influence fly behavior at a distance and that volatile, deposited and body pheromonal CHs participate in a three-step recognition of the chemical identity and social status of insects.

## Introduction

The courtship behavior of *Drosophila melanogaster* has been the subject of intense investigation [1], [2], [3], [4], [5], [6]. However, the sensory cues used to find a mate at a distance remain largely unknown. Many insects use sex pheromones to find and attract potential mates at long range [7], whereas fruit flies use both food-derived odors and pheromonal cues to gather on rotting fruits to feed and reproduce. *D. melanogaster* flies are attracted by volatile compounds produced by decaying fruits [8], [9], [10], and this effect can synergize with that of *cis*-vaccenyl acetate (*c*VA) [11], a male-specific compound transferred to the female during copulation and deposited into the food during oviposition [12], [13]. Other plant odors such as phenylacetic acid can activate a Drosophila-specific olfactory receptor (IR84a) that enhances male courtship ardor [14]. In addition to volatile long-range pheromones, some insects mark the patch on which they feed to attract (or arrest) potential mates. Food scent marking has been frequently found in Hymenoptera [15] but rarely in Diptera: two species of Tephritidae males mark individual leaves that they defend as mating territories [16], [17], whereas *Drosophila grimshawi* males deposit a long-lasting pheromone that attracts females to the lek and increases their receptivity [18], [19]. *c*VA contributes to Drosophila aggregation and could also be involved in patch marking. Moreover, *c*VA regulates other social behaviors: at higher doses, it strongly inhibits male courtship [20], [21] and stimulates female mating [22], whereas at lower doses, it promotes male aggression [23]. Because *c*VA is common to many *Drosophila* species found on the same host plants [24], adults may use additional cues to discriminate the appropriate sex partner.

In Drosophila, the only other known sex pheromones consist of long-chain hydrocarbons covering the fly cuticle (cuticular hydrocarbons, CHs) [25], [26] whose primary function is to protect insects against environmental factors [27], [28], [29], [30], [31]. *D. melanogaster* CHs show a marked sexual dimorphism: only females produce CHs with two double-bonds (dienes), while monoenes (with one double bond) are predominant in males [32], [33], [34]. Female dienes stimulate male courtship, whereas the principal male monoene, 7-tricosene (7-T), inhibits male courtship [35], [36], [37] and enhances female receptivity [38], [39]. Some quantitatively minor male monoenes may also have an important pheromonal role; e.g., 5-tricosene (5-T) may inhibit male courtship, and whereas 9- and 7-pentacosene (9-P and 7-P) could may enhance copulatory behavior [35], [40], [41]. Since As these CHs have a very reduced, or little or no volatility, they are mostly perceived by contact with the gustatory hairs located on the tarsi and proboscises of the fly [36], [42], [43], [44]. Several studies have hypothesized that Drosophila can detect at a small distance – with their olfactory organs –at small distances– – the CHs of conspecifics [38], [45], [46], [47]. However, their the volatility of CHs has never been shown, and it is not known whether *D. melanogaster* flies could can disperse CHs on the food substrate and/or into the surrounding air.

To investigate the potential dispersion of fly CHs in the environment, we used headspace Solid Phase Micro-Extraction (SPME) [48] and solvent extraction [49] coupled with GC-MS analysis. These methods allowed us to detect the presence of CHs in the air surrounding flies, on the floor of the chamber where they were kept and on their body. Because social interactions can affect CHs [50], [51], we monitored the effect of social experience on the above three CH fractions during early adult life and just after a social change in mature adults. Furthermore, we also measured the effect of sexual interactions on these three CHs fractions.

## Results

To assess the effects of “*early adult social experience*” and “*mature adult social shift*” on Drosophila CH profiles, we compared CHs in flies that varied in the following social experiences: **(** ***i*** **)** rearing conditions for the first 5 days of imaginal life and **(** ***ii*** **)** quick changes in population density at 5-days-old.

**Figure 1:**
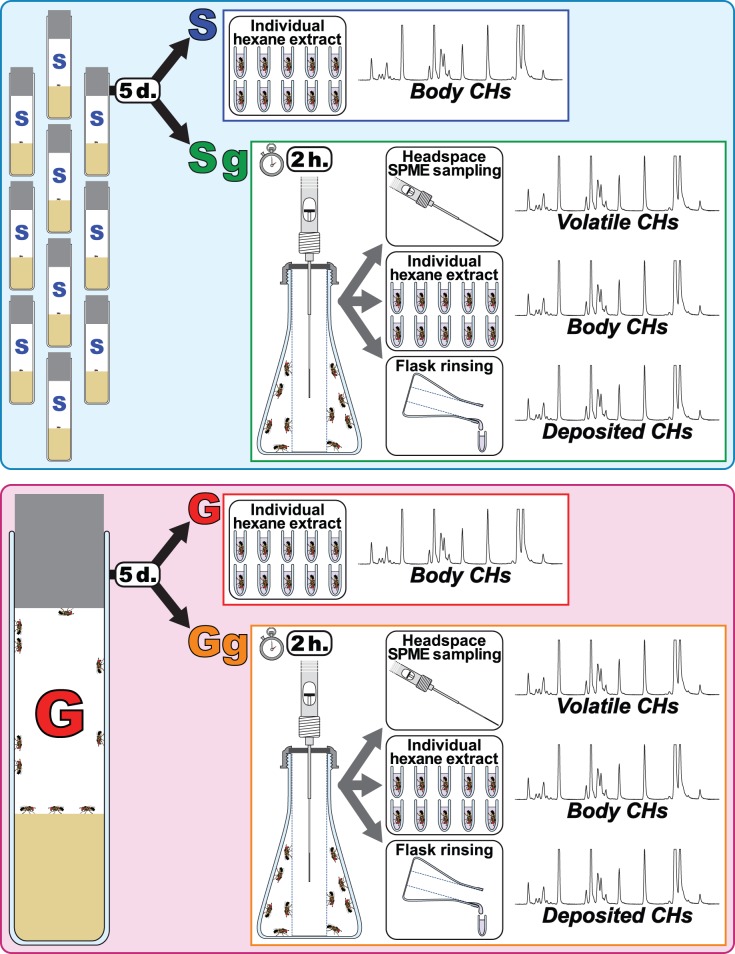
Experimental procedure for estimating the effects of different social experience on volatile, deposited and body CHs of adult flies. Five-day old singly reared flies (called “S”) were either individually hexane-extracted (body CHs) or grouped for two hours for “socialization” (groups of 10 same-sex flies  =  Sg) and thereafter sacrificed for measurements. Ten G-flies were similarly treated for measurements (“Gg” *vs* “G”). During the 2-hour socialization period, Sg and Gg flies were transferred from the large vial of 100 ml in volume, in which they spent the first five days of their adult life, to a smaller chamber (8 ml of available space) fitted with a SPME fiber. This fiber allowed us to sample volatile CHs emitted by flies during the 2-hour socialization period. Thereafter, Sg- and Gg-flies were individually hexane-extracted (body CHs), and the flask was hexane-rinsed (deposited CHs).

**Figure 2:**
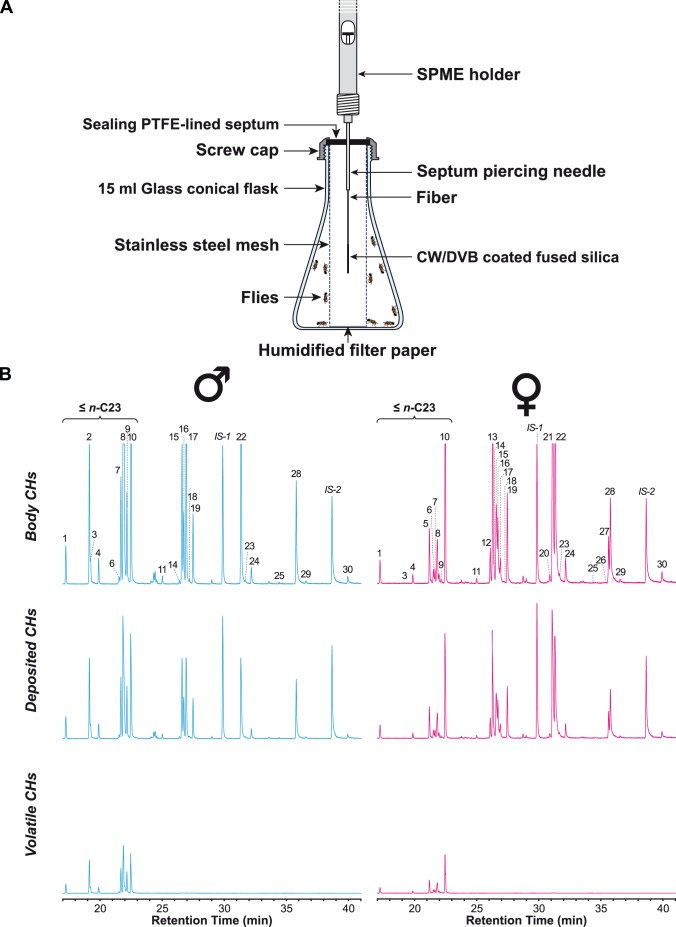
Experimental device used to sample volatile, deposited and body CHs of pooled flies and corresponding GC-MS chromatograms. **A.** Experimental device: Ten five day-old flies were pooled for 2 hours in a 15 ml glass conical flask fitted with a PTFE-lined septum. A SPME holder was clamped in place to keep the fiber at the center of a stainless steel mesh tubing (1.3 cm ID) and to prevent flies from touching the fiber. **B.** GC-MS chromatograms of body, deposited and volatile CHs in Drosophila melanogaster male and female flies. The numbers shown above the peaks refer to the peak numbers (#) listed in Table 1. IS-1 and IS-2 are the internal standards used to calculate the absolute amounts of each CH. For details about sampling and GC-MS conditions, see Materials and Methods.

Five-day old single- or group-reared flies (termed “S” and “G” flies, respectively) were either individually hexane-extracted or tested to examine the possible effect of a quick change in population density on CHs (Fig. 1). Ten S-flies were grouped for two hours for “socialization” after the 5-day isolation and then sacrificed for measurements. We termed these flies “Sg” to distinguish them from the flies that were sacrificed for measurements without experiencing any group rearing after the 5-day isolation (“S”). For comparison, 10 G-flies were similarly treated for measurements (“Gg” *vs* “G”). (Fig. 1). During the 2-hour socialization period, Sg and Gg flies were transferred from a large 100 ml volume vial in which they spent the first 5 days of adult life to a smaller chamber (15 ml), thus exposing them to an increased population density. This glass conical flask was fitted with a SPME fiber surrounded by a stainless steel mesh tubing that prevented the flies from touching the fiber (this reduced the available space to 8 ml, Fig. 2A). We used two complementary sampling tools to detect CHs in three physical fractions (Fig. 2): SPME allowed us to sample ***volatile CHs***, whereas a hexane wash was used to recover the CHs deposited by the fly on the chamber floor (***deposited CHs***) and to collect the CHs present on the fly cuticle (***body CHs***). For the sake of clarity, we only show the 30 most abundant body CHs [52] (Table 1).

**Table 1:** Major compounds detected in whole-body extracts of D. melanogaster flies.

#	Abbrev.	M	F	Compound Name
**1**	***n-*** **C21**	+	+	*n*-Heneicosane
**2**	***c*** **VA**	+		(*Z*)-11-Vaccenyl Acetate
**3**	**7-D**	+	*tr*	(*Z*)-7-Docosene
**4**	***n-*** **C22**	+	+	*n*-Docosane
**5**	**7,11-TD**		+	(*Z,Z*)-7,11-Tricosadiene
**6**	**23-Br**	+	+	2-Methyldocosane
**7**	**9-T**	+	+	(*Z*)-9-Tricosene
**8**	**7-T**	+	+	(*Z*)-7-Tricosene
**9**	**5-T**	+	+	(*Z*)-5-Tricosene
**10**	***n-*** **C23**	+	+	*n*-Tricosane
**11**	***n-*** **C24**	+	+	*n*-Tetracosane
**12**	**9,13-PD**		+	(*Z,Z*)-9,13-Pentacosadiene
**13**	**7,11-PD**		+	(*Z,Z*)-7,11-Pentacosadiene
**14**	**12-P**	+	*tr*	(*x*)-12-Pentacosene
**15**	**25-Br**	+	+	2-Methyltetracosane
**16**	**9-P**	+	+	(*Z*)-9-Pentacosene
**17**	**7-P**	+	+	(*Z*)-7-Pentacosene
**18**	**5-P**	+	+	(*Z*)-5-Pentacosene
**19**	***n-*** **C25**	+	+	*n*-Pentacosane
**20**	**9,13-HD**		+	(*Z,Z*)-9,13-Heptacosadiene
**21**	**7,11-HD**		+	(*Z,Z*)-7,11-Heptacosadiene
**22**	**27-Br**	+	+	2-Methylhexacosane
**23**	**7-H**	+	+	(*Z*)-7-Heptacosene
**24**	***n-*** **C27**	+	+	*n*-Heptacosane
**25**	***n-*** **C28**	+	+	n-Octacosane
**26**	**9,13-ND**		+	(*Z,Z*)-9,13-Nonacosadiene
**27**	**7,11-ND**		+	(*Z,Z*)-7,11-Nonacosadiene
**28**	**29-Br**	+	+	2-Methyloctacosane
**29**	***n-*** **C29**	+	+	*n*-Nonacosane
**30**	**31-Br**	+	+	2-Methyltriacontane

Peak numbers (#) correspond to the elution order of each compound (see the GC-MS trace on Figure 1B) Abbrev.  =  abbreviated names. In male and female flies, compounds were detected in quantifiable amounts (+) or in trace amounts (tr).

### Some Volatile CHs are Affected by Early Adult Social Experience

To evaluate the effect of “*early adult social experience*”, we compared the three CH fractions from the Sg- and Gg-flies (*n* = 30 S-flies and *n* = 3×10 G-flies) separately for males and females.

Although all body CHs were deposited, only a part of them were detected in the volatile fraction (Fig. 2B); this included CHs ranging from *n*-heneicosane (*n*-C21) to *n*-tricosane (*n*-C23). Among these ≤ *n*-C23 CHs, 4 were common to both sexes (*n*-C21, *n*-C22, 7-T, *n*-C23), 5 were male-specific (*c*VA, 7-D, 23-Br, 9-T, 5-T), and one was female-specific (7,11-TD).

The overall amount of body CHs (Table S1) was significantly lower in males (Sg = 1954±23 ng; Gg = 2047±71 ng) than in females (Sg = 2154±34 ng, Gg = 2281±40 ng; *K*_3df_ = 31.13, *p*≤10^−4^). Conversely, males produced more ≤ *n*-C23 compounds than females (*K*_3df_ = 89.64, *p*≤10^−4^). Deposited CHs represented 1% of the amount of body CHs (relative to both overall and ≤ *n*-C23 compounds; Table S1) and showed no sex- or early adult social experience-related variation (*K*_3df_ = 2.08, *p* = 0.61). In body and deposited CHs, ≤ *n*-C23 compounds represented 70% and 15% of the male and female total fraction, respectively. A higher amount of volatile CHs was detected in males (2–4 ng) than in females, regardless of early social experience (0.5 ng; Table S1; *K*_3df_ = 8.74, *p* = 0.01). These amounts correspond to 0.1% and 0.02% of the total body CH amount in males and females, respectively, or to 0.2% of the amount of ≤ *n*-C23 compounds in both sexes.

**Figure 3:**
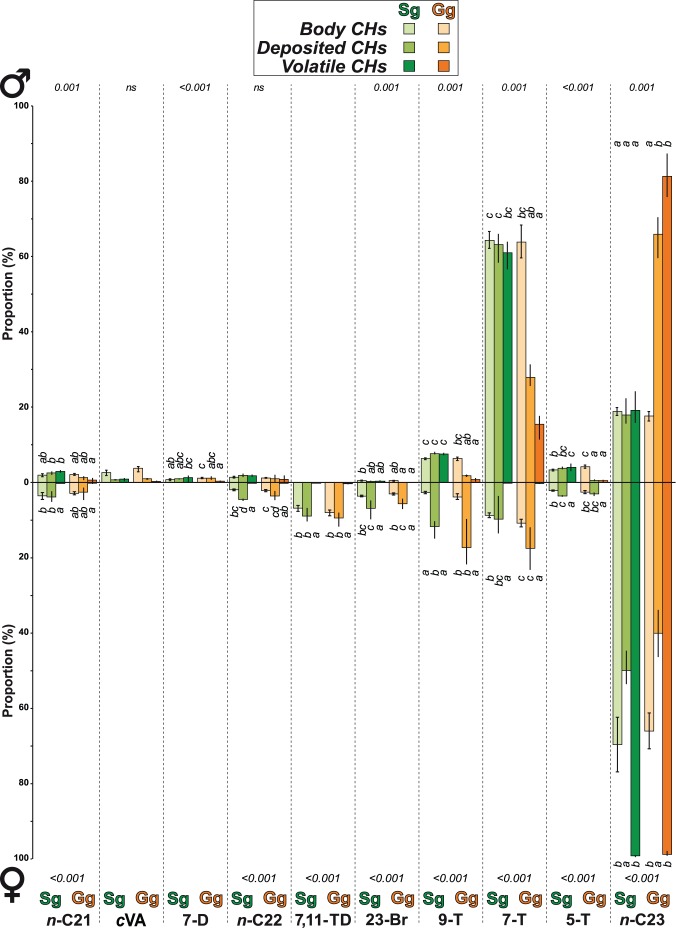
Effect of early adult social experience on ≤n-C23 compounds. During the five first days of adult life, flies were either reared singly (Sg) or grouped (Gg). The histograms represent the mean proportion (± SEM) of each compound in males (top) and females (bottom) relative to the total amount of ≤*n*-C-23 compounds in body CHs (*n* = 30), deposited CHs (*n* = 3×10 individuals), and volatile CHs (*n* = 3×10 individuals). The *p*-value shown for each CH and sex indicate the results obtained with a Kruskal-Wallis test on summed data. Different letters indicate significant differences (*p* = 0.05).

Early social experience induced clear sex- and fraction-specific effects on ≤ *n*-C23 compounds (Fig. 3). Sg males showed decreased amounts of the tricosene isomers (9-, 7- and 5-T) and increased *n*-C23 levels compared to Sg males. This reciprocal variation was found in both deposited and volatile CHs fractions –but not in the body CHs– of Sg males. In contrast, the effect of early adult social experience was reduced in Sg females, which had a slightly higher proportion of 7-T in their body CHs compared to Sg females.

### Social Change Quickly Alters Body CHs in Mature Flies

We compared CHs from same-sex S-, Sg-, G- and Gg-flies to evaluate the effect of a “*mature adult social shift* ” on the three CH fractions.

**Figure 4:**
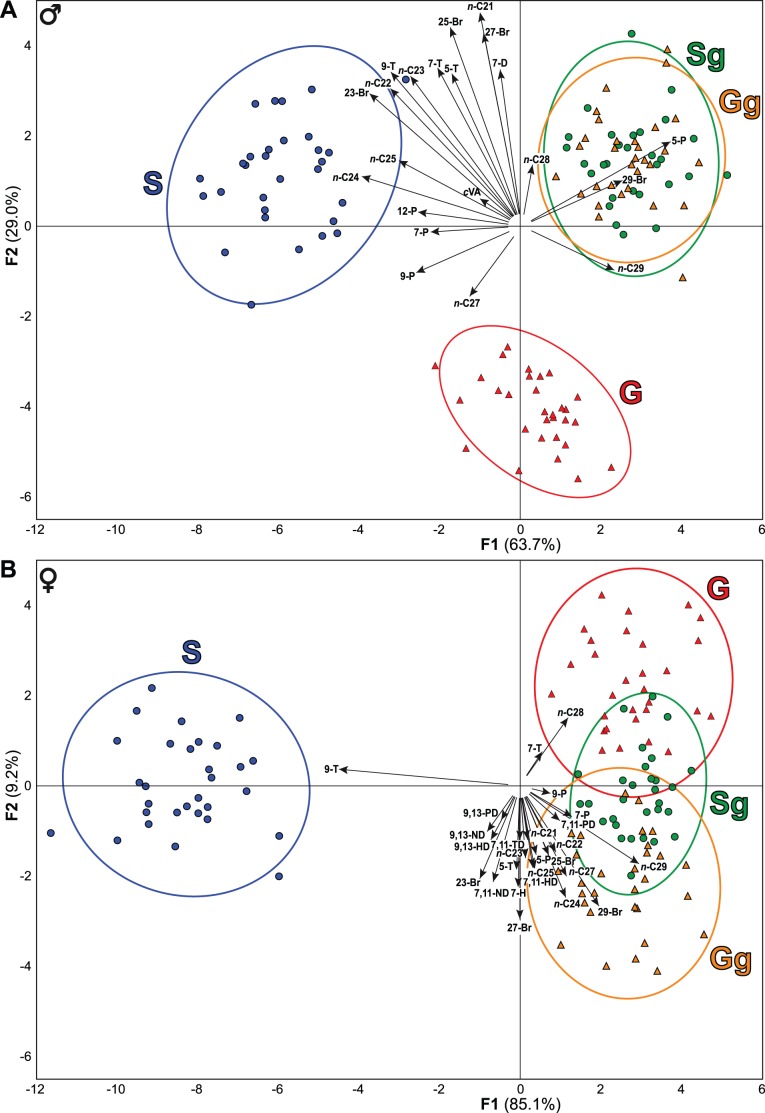
Effects of early and mature adult social experience on body CHs. To assess the effect of early adult social experience, we compared the body CHs of male (A) and female (B) flies reared either singly (S) or in groups (G). The effect of mature adult social experience was detected after the comparison of S and G flies pooled for 2 hours (Sg and Gg, respectively) in a small size chamber by groups of 10. The first plane of a stepwise discriminant analysis (DA) using the *alr*-transformed proportions of all the body CHs as quantitative variables and the social factors (S, G, Sg and Gg) as qualitative variable explains more than 90% of the total variability in both male and female flies. For all experiments, *n* = 10.

The body CH profiles strongly differed between S and G flies for both sexes, and a social shift in mature adults induced quick and sex-specific changes in their profiles. The strong initial difference between S and G males was abolished in Sg and Gg males, which showed overlapping profiles after two hours pooling (Fig. 4A). S and G females also clearly differed in their initial body CH profiles; mature adult social shift differently affected Sg and Gg females, whose profiles only partially overlapped (Fig. 4B). In particular, the body CH profile of Sg females was intermediate between those of the G and Gg females. Mature adult social shift did not affect the deposited or volatile CH fractions in either sex.

A two-way ANOVA (Table S2) allowed us to determine the effects of early adult social experience, mature adult social shift, and the interaction of these two factors on each CH and on *c*VA. Strikingly, *c*VA was the only male compound that was not affected by either condition (Table S2). Moreover, early adult social experience significantly affected 13 male CHs: the 7 most volatile CHs (*n*-C21, *n*-C22, 23-Br, 9-T, 7-T, *n*-C23 and 25-Br) were always more abundant and the 6 less volatile ones (9-P, 7-P, *n*-C27, 29-Br, *n*-C29, 31-Br) were less abundant in Sg compared to Gg males. Seventeen male CHs were significantly affected by adult social shift (and 16 by the “early experience-by-social shift” interaction) regardless of their physical or chemical properties. In females, 12 CHs were affected by early social experience, 10 were affected by adult social shift, and 8 were affected by the interaction of these factors (Table S2). However, no clear-cut pattern emerged from the CH variability in females.

### Sexual Context does not Alter the Proportions of Drosophila Volatile CHs

We also measured the effect of sexual interaction on the volatile CH fractions using 5 or 10 heterosexual pairs of S-flies (previously kept isolated) pooled in the small chamber after the 5-day isolation (SPx5 and SPx10, respectively). Their CH profiles were compared with those of Sg-males and -females (Sg-M and Sg-F, respectively) (Fig. S1). The overall amount of volatile CHs was higher in Sg-M and in SPx10 pairs (3.3±0.3 and 3.5±0.2 ng/fly, respectively) than in the Sg-F (0.8±0.1 ng/fly), whereas the amount of volatile CHs was intermediate in SPx5 pairs (2.6±0.7 ng/fly; *K*_3df_ = 25.85, *p*<10^−4^). Every ≤ *n*-C23 compound followed a parallel pattern of variation, except *c*VA and 7,11-TD.

## Discussion

Long-range pheromonal communication in *D. melanogaster* flies was initially hypothesized one century ago [53], and it received experimental support for several decades [54]. However, the only known volatile pheromone is *c*VA [12], whereas long-chain cuticular hydrocarbons (CHs), which have limited volatility, were only suspected to be detected by gustation [36], [42], [43], [44]. Several studies suggested that CHs can also be perceived at a short-distance by olfaction [25], [38], [45], [55], [56], but their volatility was never shown. Our study reveals that flies can deposit CHs on the substrate and spray CHs into the surrounding air. The dispersion of these CHs can vary both with the early and mature adult social experiences, but not with sexual interaction.

### Drosophila Adults Deposit All their CHs on the Substrate and Emit the Most Volatile Ones in the Surrounding Air

Two hours after being pooled in same-sex groups, singly raised flies (both sexes) deposited 1% of their body CHs on the substrate and sprayed 0.2% of their most volatile cuticular compounds (≤ *n*-C23) into the surrounding air.

As hydrocarbon volatility decreases with chain length [57], the selective detection of ≤ *n*-C23 compounds in the air constitutes a intriguing finding because no relationship was found between the volatile compounds detected by SPME, their amounts on the fly body, and/or their chain length. For example, we did not detect compounds with carbon chains > C23 (such as pentacosene isomers and *n*-C25), which are found in cuticular profiles (***i***) in similar amounts as tricosenes and *n*-C23 and (***ii***) in larger amounts than *n*-C21 and *n*-C22. This also contrasts with experiments using SPME sampling in cockroaches [58] and termites [59], which allows for the detection of airborne C25-C29 CHs, compounds that were nevertheless present at low concentrations on the body surface of these insects. Therefore, the differences in CH volatility between cockroaches, termites and Drosophila and between Drosophila sexes could be related to different interactions between CHs and the cuticular matrix [60].

Both deposited and volatile cuticular compounds may affect mate recognition before the first physical contact. Volatile CHs could provide a cue to guide potential sex-partners at a short distance, whereas deposited CHs could have an arresting effect and ensure that the flies remain on the marked patch of food for long durations. When resources (food or mates) are unevenly distributed, it may be an optimal strategy for animals to engage in an intensive search only on marked patches [61]. Although this context-dependent search strategy has only been described in food searching for *D. melanogaster* [62], [63], volatile and deposited pheromones are used by other species for mate finding. For example, the Ichneumonid *Venturia canescens* male is attracted by volatile compounds emitted both by the host and his female; he uses female chemicals left on the food patch to locate her [64]. *D. grimshawi* males deposit a long-lasting pheromone (of unknown nature) on the substrate that attracts conspecifics and increases mating success [18], [19]. The synergistic effect of olfactory and gustatory perception of pheromonal hydrocarbons has also been shown in the leek moth [65]. Similarly, *Drosophila* flies may be attracted at a short distance by the combined smell of food and conspecific odor [8], [9], [13], [66] and be further arrested after tasting the CHs left on food patches. However, this has never been directly demonstrated. The volatile CHs of a potential sexual partner could also be perceived by the male immediately prior to his first gustatory contact [56] and may determine whether courtship should be further pursued [1]. Male courtship behavior may be elicited by taste perception of female CHs [67] and modulated by olfactory perception [68].

The combined effect of olfaction and taste perception of pheromones on Drosophila male sexual behavior may be mediated by several olfactory receptors (Or47b, Or65a, Or67d and Or88a) that respond to fly odors [45], as well as by gustatory receptors (GR32a, Gr33a and GR68a) that are potentially stimulated by CHs [69], [70], [71]. The combined olfactory and gustatory perception of *c*VA and 7-T (with Or67d- and Gr32a-expressing neurons) may explain their effect on the modulation of male courtship and aggressive behaviors [22], [23], [51], [70], [72], [73]. The gustatory perception of female and male CHs in Gr68a- and Gr32a-expressing neurons, respectively [69], [70], requires the Gr33a co-receptor [71], and may also require Or47b- and Or88a-expressing neurons, to regulate male courtship behavior [45]. However, because the ligands of Or47b and Or88a remain unknown, these receptors may be involved in the detection of some of the volatile CHs described in the present study.

### Social Density Change in Mature Adults Alters Fly CHs More Strongly than Early Adult Social Experience

Variations in the social environment can affect Drosophila male courtship and aggressive behaviors cuticular profiles [37], [50]. A change of the social structure also induces rapid CH variations in crickets [74]. Moreover, our current data reveal a relationship between the social experience of fruit flies and their volatile pheromone profile.

In our study, we compared the effects induced by two types of social experience: (***i***) a long-lasting exposure during early adult development and (***ii***) a rapid shift during mature adult life. Our results indicate that both types of social experience can affect the CHs found on the fly body, released in the air, and released onto the substrate. Social experience generally induced a greater effect in males than in females. Moreover, ≤ *n*-C23 compounds were present in higher amounts –and other CHs were present in lower amounts– in isolated males than in group-reared males. Our results support the hypothesis of a previous study that the *D. melanogaster* male cuticular profile is not only determined by the genotype or by the environment [50] but also by individual experience in males and –to a lesser extent– in females. However, these studies diverged in the following aspects: in the former study, most fly body CHs (except 9-T, *n*-C24 and 29-Br) were significantly affected by the group composition (with the highest effect on *n-*C27 and *c*VA), whereas 7-T showed the highest amount of variability related to the social-by-environment interaction [50]. In contrast, we found that *c*VA was the only male compound that does not vary with social experience (supporting a recent finding [51]) and that 7-T was only slightly affected by social experience. These discrepancies may be explained by the different genotypic structures of the populations used in both studies (wild-type males were either mixed [50] or not [the present study] with mutant males) and/or the variety of methods used to analyze CHs. Moreover, only volatile and deposited CHs were measured in our study. A genotype-by-experience interaction has previously been shown to differentially affect male courtship behavior, and these effects may be related to the CH profile [75].

The adjustment of the three CH fractions (body, deposited, and volatile) as a response to social experience differed between the sexes: males and females were similarly affected by early adult exposure, while males were more affected than females by the mature adult shift. This male-specific “late” effect may be caused by the frequent aggressive interaction between mature males that is related to a pheromonal change [23], [51], [73], [76], [77], [78], [79]. Social experience during early adult life induces different effects; it reduces male aggressive or dominant behavior via chronic exposure to *c*VA [51], 7-T and/or 7-P during the first 24 hours of adult life [80]. This indicates that 7-T and 7-P act in a hierarchical manner with *c*VA to regulate Drosophila male aggressive and dominance behavior [73]. Therefore, early adult exposure of grouped males to *c*VA may reduce their emission of 7-T (and other volatile CHs), which, in turn, could reduce male-male aggressive behavior.

Strikingly, early adult experience induced reciprocal effects on the proportions of two sets of male CHs; the tricosene isomers decreased, whereas *n-*tricosane increased. This effect was found in both deposited and volatile CHs compared to body CHs. Because the level of these two sets of CHs is linked by the biosynthetic activity of enzymes such as Δ-9 desaturase (coded by the *desat1* gene), social experience may interfere with the expression of this gene to regulate the level and emission of some CHs [34], [50]. However, this does not explain why social experience differentially affected the deposited and volatile CHs and not the body CHs.

### Sexual Context does not Alter the Amounts of Volatile CHs

In contrast to social interaction, sexual interaction had a limited effect on CHs; it did not change the level of volatile compounds, and the variation of most volatile ≤ *n-*C23 compounds (except 7,11-TD) was proportional to the number of males involved in the group. This result is in contrast to that obtained in our previous study [52] that showed that mating interaction can rapidly alter body CHs either by a reciprocal mechanical transfer of male predominant monoenes (plus *c*VA) to the female cuticle and of female dienes to the male cuticle or by the unilateral post-mating variation of some CHs previously identified as putative *ur*-pheromones that potentially induce a non-species specific sexual excitation [81]. The differential interactions between CHs and the cuticular matrix [60] could be the basis of the observed differences between our previous and current findings on the effect of sexual context on body and volatile CHs.

In summary, our data suggest that Drosophila CHs participate in at least three successive sequences of pheromone perception during mate choice and courtship behavior. First, flies attracted by food odors combined with *c*VA [8], [9], [10], [11], [13], [14], [66] may be arrested on the food patch marked with deposited CHs. Second, volatile CHs could facilitate the localization, at a short-distance, of potential mates and/or enhance some preliminary steps of male and female courtship [35], [38], [56]. Third, body CHs could be perceived by contact to enhance or inhibit male courtship behavior [33], [35], [36], [37], [67]. Female CHs have previously been shown to act in three sequential reproduction-related aspects: mate choice, copulation duration and the sex-ratio of the progeny [82].

Our next challenge will be to better understand the behavioral role of the deposited and volatile CHs of Drosophila flies during their social and sexual interaction and to map the neural structures involved in their detection.

## Materials and Methods

### Fly Husbandry

Flies of the wild-type strain Dijon 2000 (Di2 [37]) were raised and tested at 24±0.5°C and 65±5% relative humidity on a 12∶12 h L:D cycle. Stocks were maintained in 150 ml glass vials containing 50 ml of yeast/cornmeal/agar laboratory medium.

### General Methods

Each morning, 1-h-old flies were sexed under light CO_2_ anesthesia and kept in fresh-food vials either singly (S) or in groups of 10 (or 5) flies (G) of the same sex. At 5 days old, S and G flies were either individually hexane-extracted or grouped either in same-sex or in heterosexual groups. To examine the possible effect of social interactions on CH profiles, 10 S-flies were grouped for 2 hours for “socialization” after the 5-day isolation and thereafter sacrificed for measurements. We termed these flies “Sg” to distinguish them from the flies that were sacrificed for measurements without experiencing any group rearing after the 5-day isolation (“S”). For comparison, 10 G-flies were similarly treated before measurements (“Gg” *vs* “G”). During the 2-hr socialization period, the flies were transferred from a large 100 ml volume vial in which they spent the prior 5-days to a small 15 ml volume chamber to expose them to a high population density (Fig. 1). This 15 ml glass conical flask was fitted with a PTFE-lined septum (Fig. 2A). A Supelco SPME holder was clamped in place in such a way that the SPME fiber was at the center of a stainless steel mesh tubing (1.3 cm ID) to prevent flies from touching the fiber. In this device, flies had 8 ml of available space. To examine the possible effect of sexual interactions on volatile CH profiles, S-flies were gathered in groups of 5 or 10 heterosexual pairs (SPx5 and SPx10, respectively) for two hours in the same device.

As in a previous study [52], we used a StableFlex fiber covered with carbowax/divinylbenzene (CW/DVB, 70 µm, Supelco, St Quentin-Fallavier, France). Prior to each sampling, the fiber was conditioned for 30 min at 230°C in the injection port of a gas chromatograph (GC). According to the type of experiment, we collected volatile chemicals from the flask headspace using SPME (volatile CHs), we rinsed the flask with hexane (deposited CHs), and/or we individually extracted the fly CHs with hexane (body CHs).

### Cuticular Hydrocarbon Sampling

#### Volatile CHs

The SPME sampling recovery rates were first determined using various amounts (500 ng, 1, 5 and 10 µg) of synthetic compounds (*n*-C21, *n*-C22, *n*-C23, *n*-C24, *n*-C25, *c*VA, 9-T) that contribute to the cuticular profile of *D. melanogaster*. Ten microliters of a hexane mixture that included all compounds was deposited on a 1 cm^2^ glass microscope lamella. The solvent was allowed to evaporate for 1 min at room temperature before the lamella was introduced in the flask, and the volatiles collected for 2 hours. To sample volatile CHs of fly groups, we collected the headspace volatile chemicals for 2 hours after fly pooling (no change was noted after 6-hours of sampling except for traces of 25-Br).

After retraction of the fiber, the SPME device was introduced for 1 min into the injection port of a GC-MS to allow the compounds to desorb. We used a QP2010 Shimadzu GC-MS apparatus in splitless mode fitted with a VF-1ms fused silica capillary column (20 m×0.15 mm ID, 0.15 µm film thickness; Varian, The Netherland). The column was held isothermally at 140°C and then programmed at a rate of 3°C/min to 300°C. Helium was used as a carrier gas at a linear velocity of 47 cm/sec. The injector port was set at 280°C. The mass spectrometer was operated at 70 eV and scanning was performed from 29 to 600 amu at 0.5 scans/sec. The injection split was opened 1 min after injection. The detected components were identified using their Kovats’ indices [83]. The fragmentation patterns and diagnostic ions of the components were then compared with the NIST/EPA/NIH library, our own mass-spectrum library and previously published Drosophila CHs. The linearity and the calibration of the detector response were tested by injection of 1 µl of hexane solutions of the corresponding compounds over the range of 0.1, 0.5, 10 and 100 ng of *n*-C21, *n*-C22, *n*-C23, *n*-C24, *n*-C25, *c*VA and 9-T. We used the resulting calibration curves to calculate the absolute amount of the volatile CHs.

#### Body CHs

After headspace SPME sampling of volatile CHs, the flies were cold anesthetized (5 min at −20°C) and individually plunged, at room temperature, for 5 min into vials containing 30 µl hexane with 100 ng *n*-C26 and 100 ng *n*-C30 as internal standards (IS-1 and IS-2, respectively) following standard procedures [49]. IS-1 and -2 compounds were chosen because Di2 flies of both sexes lack these alkanes. After fly removal, the extracts were stored at −20°C until their analysis using the same GC-MS conditions employed for the volatile CHs. IS-1 and IS-2 were used to estimate the absolute amount of all the body CHs.

#### Deposited CHs

After volatile CHs sampling and removal of the flies, we rinsed the flask with 1 ml hexane containing 100 ng each of IS-1 and IS-2 to collect the deposited CHs. The hexane rinse was reduced to approximately 30 µl under a light N_2_ stream, and GC-MS analyses were performed with similar conditions as those previously described. IS-1 and IS-2 were used to estimate the absolute amount of all the deposited CHs.

### Statistical Procedures

Statistics were performed using XLSTAT 2011 [84]. To compare the 3 CH fractions (body, deposited and volatile), we used the Kruskal-Wallis test with a Monte-Carlo resampling of the N values (10^5^ simulations) to compute the associated *p*-value. Significant Kruskal-Wallis tests were followed with Conover-Iman’s multiple pairwise comparisons (two-tailed with Bonferroni correction).

To assess the effect of social context on the cuticular profiles, the relative amounts of body CHs were transformed with the additive log-ratio transformation (*alr*) [85] using CoDaPack (v2.01.1; [86]); the zero values were substituted by 10^−6^ values and the divisor was taken to be the proportion of 31-Br. For each sex, we carried out a stepwise (forward; entry threshold: *p* = 0.05; removal threshold: *p* = 0.10) discriminant analysis (DA) using the *alr*-transformed CH proportions as quantitative variables and the social context as a qualitative variable. The effects of early adult social experience and mature adult social shift, and their interaction, were tested using a two-way ANOVA after a Box-Cox transformation (optimized Lambda) of the body CH proportions.

## Supporting Information

Figure S1 **Absolute amounts of the ≤n-C23 volatile compounds in different groups of flies.** Compounds were sampled in same sex groups (x10) of males (Sg-M) and females (Sg-F) and in groups of 5 and 10 heterosexual pairs (SPx5 and SPx10, respectively). The amounts are expressed in ng/individual as the mean (SEM or range). Letters indicate significant differences (Kruskal-Wallis test). For compound names, see Table1. *n* = 5×10 or 20 individuals.
(EPS)

Table S1 **Amount of all compounds and of ≤n-C23 compounds in body, deposited and volatile CH fractions from 5-day-old S- and G-flies grouped in same-sex groups of 10 individuals for a two hour “socialization” period in a small 15 ml vial (Sg- and Gg-flies).** The amounts are expressed as the mean (SEM or range) in units of ng/individual. Letters indicate significant differences (Kruskal-Wallis test, *p* = 0.05). **^1^**: As volatile fractions consisted of only ≤*n-*C23 compounds, their amount values were tested only once.
(EPS)

Table S2 **Results of the two-way ANOVA using the Cox-Box transformed CH proportions of all the cuticular compounds as quantitative variables and the two social changes (early, mature), in male (A) and female (B).** HSD: letters indicate significant differences (Tukey’s HSD test, *p* = 0.05). For compound names, see Table1. (*n* = 5×10 or 20 individuals).
(EPS)
